# The impact of thioredoxin reduction of allosteric disulfide bonds on the therapeutic potential of monoclonal antibodies

**DOI:** 10.1074/jbc.RA119.010637

**Published:** 2019-11-14

**Authors:** Shalom A. Gurjar, Jun X. Wheeler, Meenu Wadhwa, Robin Thorpe, Ian Kimber, Jeremy P. Derrick, Rebecca J. Dearman, Clive Metcalfe

**Affiliations:** ‡ Division of Biotherapeutics, The National Institute for Biological Standards and Control, Blanche Lane, South Mimms, Potters Bar, Hertfordshire EN6 3QG, United Kingdom; § Division of Technology Development and Infrastructure, National Institute for Biological Standards and Control, Blanche Lane, South Mimms, Potters Bar, Hertfordshire EN6 3QG, United Kingdom; ¶ Lydia Becker Institute of Immunology and Inflammation, School of Biological Sciences, Faculty of Biology, Medicine and Health, Manchester Academic Health Science Centre, University of Manchester, Manchester M13 9PT, United Kingdom

**Keywords:** allosteric regulation, thioredoxin (Trx), monoclonal antibody, redox regulation, disulfide, antibody-dependent cellular cytotoxicity (ADCC), biotherapeutic, functional effects, safety and efficacy, disulfide bond, complement-dependent cytotoxicity (CDC)

## Abstract

Therapeutic mAbs are used to manage a wide range of cancers and autoimmune disorders. However, mAb-based treatments are not always successful, highlighting the need for a better understanding of the factors influencing mAb efficacy. Increased levels of oxidative stress associated with several diseases are counteracted by the activities of various oxidoreductase enzymes, such as thioredoxin (Trx), which also reduces allosteric disulfide bonds in proteins, including mAbs. Here, using an array of *in vitro* assays, we explored the functional effects of Trx-mediated reduction on the mechanisms of action of six therapeutic mAbs. We found that Trx reduces the interchain disulfide bonds of the mAbs, after which they remain intact but have altered function. In general, this reduction increased antigen-binding capacity, resulting in, for example, enhanced tumor necrosis factor (TNF) neutralization by two anti-TNF mAbs. Conversely, Trx reduction decreased the antiproliferative activity of an anti-tyrosine kinase-type cell-surface receptor HER2 mAb. In all of the mAbs, Fc receptor binding was abrogated by Trx activity, with significant loss in both complement-dependent cytotoxicity and antibody-dependent cellular cytotoxicity (ADCC) activity of the mAbs tested. We also confirmed that without alkylation, Trx-reduced interchain disulfide bonds reoxidize, and ADCC activity is restored. In summary, Trx-mediated reduction has a substantial impact on the functional effects of an mAb, including variable effects on antigen binding and Fc function, with the potential to significantly impact mAb efficacy *in vivo*.

## Introduction

Disulfide bonds are covalent bonds formed between the thiol groups of two cysteine amino acid residues in a protein or a protein complex. They impart mechanical stability to protein domains, such as the immunoglobulin domain. They are particularly prevalent in proteins that reside on the plasma membrane of cells or are secreted extracellularly. The extra stability conferred by disulfide bonds protects proteins from degradation by proteases and the large pH changes that occur in the harsh extracellular environment (1). Although disulfide bonds are generally thought to be stable, in some proteins, certain disulfide bonds termed “allosteric” disulfide bonds are labile and susceptible to post-translational reductive cleavage, which can modulate their activity (2). The reduction of labile disulfide bonds is carried out by thiol oxidoreductase enzymes, such as thioredoxin (Trx),^3^ protein disulfide isomerase, ErP5, and ErP57. Trx is a small (12-kDa) redox-active protein essential for the survival of both prokaryotic and eukaryotic cells (3). Its active site contains a reactive dithiol within a cysteine-glycine-proline-cysteine motif that can donate electrons to reduce susceptible disulfide bonds. The resulting oxidized Trx is then regenerated by thioredoxin reductase 1 (Tr1), which uses NADPH as a source of electrons (4). Trx is up-regulated in response to oxidative stress (5, 6) and is secreted into the extracellular environment in quickly dividing cells, such as activated immune cells (7) and cancers (8). Multiple studies show that thiol oxidoreductase enzymes secreted extracellularly during immune activation increase the abundance of free thiols derived from reduced disulfide bonds in surface membrane proteins (9, 10). Due to the potential for cell-surface proteins to modulate cellular function, it has been demonstrated that this has a profound effect on immune system regulation (11). Recently, a MS-based technique has been developed that can identify labile disulfide bonds in immune cell-surface proteins reduced by Trx and protein disulfide isomerase *in vitro* and on the surface of mouse splenocytes *in vivo* after lipopolysaccharide-induced acute endotoxemia (12). Over 80 proteins with labile disulfide bonds were identified; many have been shown to be allosteric disulfide bonds that control protein function. This usually manifests itself as modulation of ligand binding, as observed in the key immune proteins interleukin-2 (IL-2) receptor (13), CD44 (14), and LYVE-1 (15).

As well as playing a role in immune activation, dysregulation of labile disulfide bonds can contribute to disease. Elevated levels of extracellular thiol oxidoreductase enzymes also correlate with pathological levels of oxidative stress. High Trx levels have been measured in tissues derived from various cancers, including gastric, lung, cervical, pancreatic, and breast cancer (5). Mouse studies have revealed that both the size and extent of metastasis of tumors correlate with the level of Trx expression (16, 17), as does the resistance of multiple breast cancer cell lines to the chemotherapeutic drugs doxorubicin and cisplatin (18).

In addition to cancer, Trx is involved in the etiology and continued pathology of chronic inflammatory diseases, such as rheumatoid arthritis (RA), which primarily affects joints and can result in cartilage and bone degradation (19). In inflammatory environments, such as an RA joint, Trx is secreted by activated lymphocytes. The elevated levels of Trx, which can be detected in the blood and synovial fluid of RA patients, can promote the growth of fibroblast-like synoviocytes (FLSs) within the joints of RA patients. These hyperproliferating FLS cells release inflammatory cytokines, perpetuating inflammation. Furthermore, FLS cells can migrate to, and cause pathology in, other joints. Thus, Trx contributes to disease progression (20).

**Figure 1.**
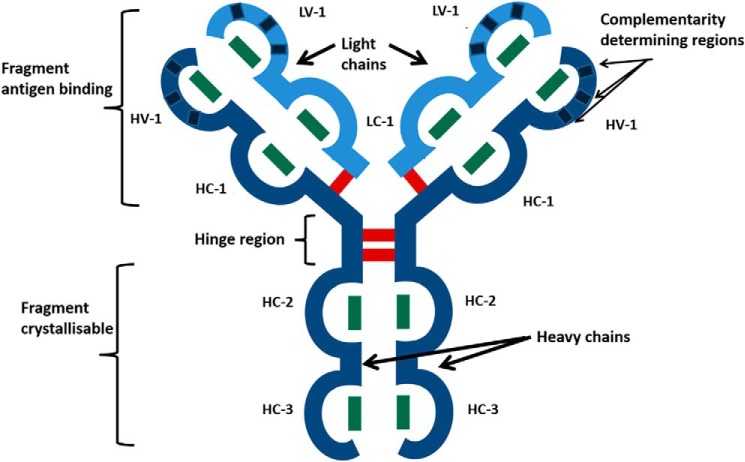
**Representation of IgG1 structure.** *Red* and *green lines*, inter- and intrachain disulfide bonds, respectively. *V*, variable domain; *C*, constant domain; *H*, heavy; *L*, light.

mAb therapies have revolutionized the treatment of both cancers and autoimmune disorders. Their success over the last 20 years is due largely to their ability to bind specifically and with a high affinity to almost any desired target antigen. Most approved therapeutic mAbs are of the IgG1 subclass, and their sequences are either chimeric mouse/human, humanized, or fully human. All contain 32 conserved cysteine residues forming 16 conserved disulfide bonds (Fig. 1). Both heavy and light chains consist of Ig-like domains, each of which contains an intrachain bond. Interchain disulfide bonds are also present: one between each heavy and light chain and two between the two heavy chains located at the hinge region (21). The cost-effective, efficient, and safe manufacture of mAbs, which is currently achieved using Chinese hamster ovary (CHO) cells, is critical to the bioprocessing industry (22). Owing to advances in batch culture production, product yields can be remarkably high; currently for CHO cells, yields reach up to 12 g/liter, an increase from the upper limit recorded in 2004, of 5 g/liter (23). However, the use of accelerated harvesting techniques can compromise the integrity of the final product. Several studies have shown that thiol oxidoreductase enzymes are released into the supernatant upon cell lysis due to the use of excessive mechanical force, resulting in unwanted disulfide bond reduction of mAbs (24). Whereas mAb reduction can be detected during quality control and, importantly, prior to product release, the economic consequences of the resulting failed batch can be severe (25).

Although mAb therapies have improved the treatment of disease, they are not 100% effective. Many patients do not initially respond or lose response over time, often due to the patient developing anti-drug antibodies as a result of unwanted immunogenicity. For example, ∼50% of patients with RA either do not respond or lose response to the anti-tumor necrosis factor (TNF) mAb infliximab (26). Trastuzumab, a mAb used to treat breast cancer, is only effective in ∼35% of patients, with ∼70% eventually becoming resistant (27). Furthermore, ∼30–60% of non-Hodgkins lymphoma patients are resistant to rituximab (28). Recently, tregalizumab, an anti-CD4 mAb that activates regulatory T cells developed to treat RA, failed clinical trials due to low efficacy (29). Data from clinical trials had shown that CD4 down-modulation, a measure of regulatory T-cell activation, was significantly lower in RA patients compared with normal subjects. *In vitro* studies showed that the lack of efficacy was due to Trx selectively reducing a disulfide bond within CD4 near the binding site of tregalizumab. This consequently impairs the binding of tregalizumab to CD4, demonstrating the ability of Trx secreted in an inflammatory environment to modulate antibody/ligand interactions.

Given that therapeutic mAbs contain many disulfide bonds that can be reduced by Trx during manufacture and potentially *in vivo*, and that they deliver their *in vivo* therapeutic effects in Trx-rich extracellular environments, we thought it pertinent to examine the effect of Trx on the structure and function of a selection of clinically important therapeutic mAbs. Previous work has demonstrated that human IgG1 and, to a lesser extent, IgG2 mAbs are susceptible to chemical reduction with DTT (30). The current study extends these findings to demonstrate the impact of the more physiological Trx-mediated reduction on the IgG1 therapeutic mAbs infliximab and adalimumab, which both neutralize TNF; rituximab and ofatumumab, which both target CD20; and trastuzumab and cetuximab, which target epidermal growth factor receptors HER2 (human epidermal growth factor receptor 2) and HER1, respectively. In addition to assessing their biophysical characteristics and antigen binding, we focus in detail on the primary mode of action of the mAbs.

## Results

### Therapeutic mAbs are substrates for Trx, and they contain labile disulfide bonds

**Figure 2.**
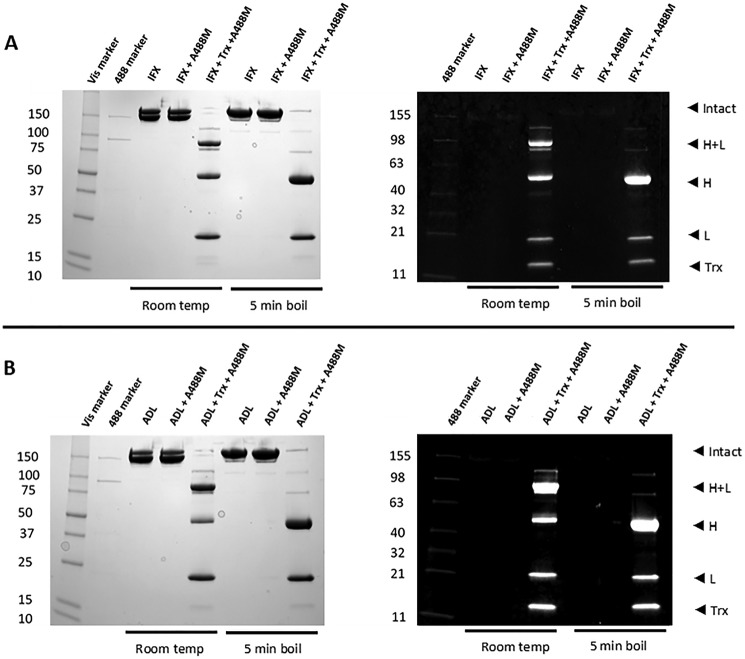
**Nonreducing SDS-PAGE of infliximab (*IFX*) (*A*) and adalimumab (*ADL*) (*B*) before and after reduction with Trx and labeling of cysteines from reduced disulfide bonds with Alexa-488-maleimide.** Gels were visualized at 488 nm to probe cysteine labeling (*right*) and Coomassie-stained for total protein (*left*). 12 μg of protein was loaded onto each well in 20 μl of nonreducing sample-loading buffer; one set of samples was boiled for 5 min prior to loading. *H*, heavy; *L*, light.

To study the effect of the Trx system *in vitro*, therapeutic mAbs infliximab, adalimumab, rituximab, trastuzumab, ofatumumab, and cetuximab were treated with Trx using conditions previously used to reveal a labile disulfide bond in interleukin-4 (31). Free cysteines liberated from the reduction of labile disulfide bonds were labeled with a fluorescently conjugated cysteine thiol-reactive maleimide reagent, Alexa-488-maleimide (A488M), and the samples were separated by nonreducing SDS-PAGE. A detailed analysis for infliximab (Fig. 2*A*) and adalimumab (Fig. 2*B*) reveals that both mAbs are reduced by Trx. In both cases, Trx reduces the heavy-light and heavy-heavy interchain disulfide bonds as the mAbs run as a combination of heavy-chain/light-chain monomers (band at ∼75 kDa) and free heavy (band at ∼50 kDa) and light chains (band at ∼25 kDa). Boiling is required to fully dissociate the Trx-reduced mAbs into free heavy and light chains, suggesting that they still retain some quaternary structure after reduction of the interchain disulfide bonds. Visualization of the A488M labeling reveals that, as expected, intact infliximab and adalimumab contain no free cysteines; however, after Trx reduction, both the heavy and light chains are labeled. More intense bands on the heavy chains suggest that more free cysteines are liberated upon reduction than in the light chains. Rituximab, trastuzumab, ofatumumab, and cetuximab were also reduced by Trx in a similar way as shown by Coomassie-stained nonreducing SDS-PAGE (Fig. S1). This shows that IgG1 mAbs are substrates for Trx and contain labile disulfide bonds.

**Figure 3.**
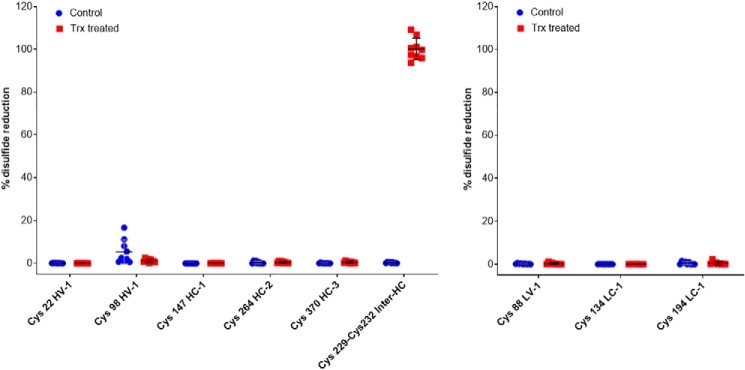
**Quantitative MS showing the percentage reduction of each disulfide bond of infliximab after treatment with Trx.** *Left panels*, heavy chains; *right panels*, light chains.

Fluorescent labeling provides a qualitative picture of disulfide reduction, but it does not reveal the identity of liberated cysteines. To determine which intra- and/or interchain disulfide bonds in the IgG-like domains are reduced, a more in-depth quantitative MS analysis was performed on infliximab to determine the percentage reduction of each disulfide bond by Trx. Infliximab was reduced by Trx as above, and liberated cysteines resulting from reduced disulfide bonds were labeled with iodoacetamide (IAA). The mAbs were then trypsinized and peptides sequenced by LC-MS-MS. A 100% reduced sample of each mAb, with all of the cysteines fully labeled with IAA, as well as a nonreduced but IAA-treated control sample were also sequenced. Peptides containing cysteines from each of the disulfide bonds as well as control peptides that contained no cysteine residues were selected (Table S2), and precursor ion areas were extracted from the MS data. To determine a measure of disulfide bond reduction, the area of the cysteine peptide in the IAA control or Trx sample was expressed as a percentage of the area of the same cysteine peptide in the 100% reduced sample (Equation 1; calculations shown in the supporting material). The ratio of a cysteine peptide to a control peptide in the same sample should be similar regardless of column loading and trypsin digestion efficiency. This approach of using internal reference peptides to normalize data has been used previously (32). Mass spectroscopy confirmed 100% reduction of inter-heavy chain bonds and, importantly, revealed that no intrachain disulfides were reduced. (A small amount of reduction was observed for cysteine 98 in the control sample; however, this seems to be an artifact of several of the injections due to the weak ionization of this peptide. Cysteine 22, which forms the other half of the HV disulfide bond, is a much stronger peptide and shows no such reduction (Fig. 3).) As all of the mAbs in the study are of the IgG1 isotype, it is reasonable to assume that reduction patterns will be similar for all, supporting the conclusions drawn from SDS-PAGE analysis (Fig. 2).

**Figure 4.**
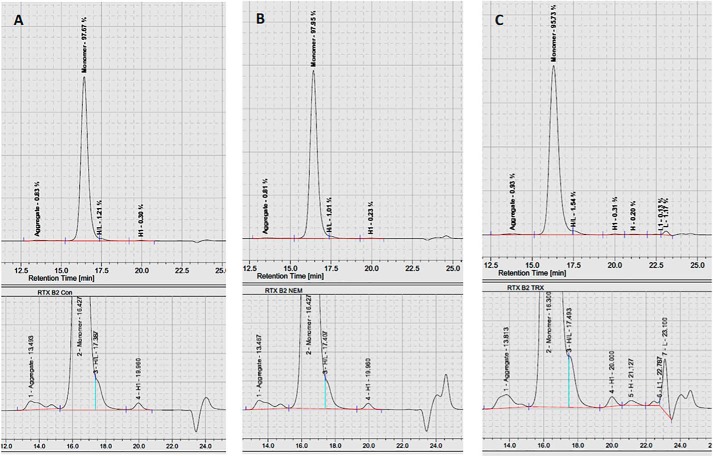
**SEC-HPLC of rituximab nonreduced control (*A*), NEM-treated rituximab (*B*), and Trx-reduced and NEM-alkylated rituximab (*C*).** Percentage abundance values for aggregate, monomer (fully intact), and fragments along with retention time are summarized in Table 1. The profile (absorbance at 280 nm *versus* retention time) is representative of three independent repeat experiments performed for the mAbs.

To determine whether mAb quaternary structure is maintained when interchain disulfide bonds are reduced by Trx, the mAbs were analyzed by SEC-HPLC, which is routinely used to quantify mAb fragmentation and aggregation. Representative chromatograms of rituximab are shown in Fig. 4, and the data from all of the mAbs are summarized in Table 1. Importantly, Trx reduction did not increase the amount of aggregated material in the samples, which remained below the limit of detection (<1%) in all samples; overall, the mAbs stayed intact (Table 2). After Trx reduction, a slight decrease in fully intact mAb was observed and was determined to be statistically significant for some mAbs, but, importantly, >95% fully intact mAb was retained. Interestingly, the Trx-reduced samples did, however, elute slightly earlier, indicating that they had a slightly increased hydrodynamic radius after reduction indicative of a small structural perturbation.

**Table 1:** **SEC-HPLC purity profile of reduced mAbs with percentage abundance and retention times** Untreated control, NEM-only-treated, and Trx-treated mAbs were analyzed using SEC-HPLC. Presented values are mean percentage abundances for aggregate, monomer (fully intact), and fragments with S.D., derived from analyses of three independent batches for each mAb. ND, not determined.

Antibody and treatment	Aggregate		Monomer		Heavy/Light		Heavy		Light	
	Relative area	Retention time	Relative area	Retention time	Relative area	Retention time	Relative area	Retention time	Relative area	Retention time
	%	*min*	%	*min*	%	*min*	%	*min*	%	*min*
**Rituximab**										
Control	1.2 ± 0.7	13.60	97.5 ± 0.5	16.37	1.1 ± 0.1	17.41	0.3 ± 0.0	19.97	ND	
NEM	0.9 ± 0.1	13.51	98.1 ± 0.3	16.41	0.7 ± 0.3	17.62	0.3 ± 0.0	19.96	ND	
Trx	0.9 ± 0.0	13.70	95.5 ± 0.4	16.32	1.5 ± 0.3	17.55	0.8 ± 0.4	19.99	1.3 ± 0.4	22.44
**Ofatumumab**										
Control	1.4 ± 0.8	14.00	96.2 ± 0.5	16.58	1.1 ± 0.2	17.75	0.3 ± 0.0	20.12	ND	
NEM	1.1 ± 0.4	14.00	96.8 ± 1.1	16.58	1.0 ± 0.1	17.61	0.2 ± 0.0	20.12	0.2 ± 0.3.	22.45
Trx	0.8 ± 0.1	13.88	94.3 ± 1.6	16.43	2.0 ± 0.7	17.64	0.8 ± 0.4	20.13	1.2 ± 0.8	22.47
**Cetuximab**										
Control	0.6 ± 0.3	13.80	97.8 ± 0.3	16.24	1.4 ± 0.4	17.35	0.4 ± 0.1	19.84	ND	
NEM	0.5 ± 0.4	13.75	98.1 ± 0.2	16.18	1.1 ± 0.2	17.31	0.3 ± 0.1	19.74	ND	
Trx	0.4 ± 0.3	13.66	95.4 ± 0.1	16.02	2.8 ± 0.1	17.37	0.7 ± 0.1	19.72	0.7 ± 0.5	22.49
**Trastuzumab**										
Control	1.0 ± 0.4	13.83	98.4 ± 0.2	16.28	0.8 ± 0.3	17.36	0.1	19.92	ND	
NEM	1.9 ± 2.1	13.91	97.5 ± 1.6	16.37	0.7	17.32	0.1	19.97	ND	
Trx	0.7	13.83	96.8 ± 0.7	16.26	1.2 ± 0.4	17.46	0.4 ± 0.1	19.99	1.1 ± 0.8	22.45
**Infliximab**										
Control	0.1 ± 0.1	13.49	98.7 ± 1.7	16.38	0.6 ± 1.1	17.45	0.1 ± 0.0	20.17	ND	
NEM	0.3 ± 0.0	13.48	99.6 ± 0.1	16.38	ND		0.1 ± 0.1	19.72	ND	
Trx	0.5 ± 0.0	13.54	95.9 ± 0.7	16.07	2.1 ± 0.3	17.30	0.8 ± 0.2	20.09	0.8 ± 0.2	22.45
**Adalimumab**										
Control	0.5 ± 0.1	13.71	98.7 ± 0.1	16.15	0.7 ± 0.1	17.13	0.2 ± 0.0	19.77	ND	
NEM	0.5 ± 0.0	13.70	98.5 ± 0.1	16.15	0.8 ± 0.0	17.14	0.2 ± 0.0	19.77	ND	
Trx	0.5 ± 0.0	13.56	96.0 ± 0.4	15.92	1.8 ± 0.2	17.30	1.0 ± 0.1	19.71	0.7 ± 0.1	22.46

**Table 2:** **Summary of the statistical significance for the difference in the percentage of intact mAb *versus* controls, carried out using two-tailed *t* test analysis**

Intact mAb	Statistically different? Yes or No (*p* value)	
	Trx-reduced *versus* untreated control	NEM-treated control *versus* untreated control
Rituximab	Yes (0.049)	No (0.30)
Ofatumumab	No (0.16)	No (0.21)
Cetuximab	Yes (0.0058)	No (0.27)
Trastuzumab	No (0.063)	No (0.45)
Infliximab	Yes (0.013)	No (0.37)
Adalimumab	Yes (0.0072)	No (0.22)

Having confirmed that quaternary structure and intrachain disulfide bonds were unaffected by Trx reduction of interchain disulfide bonds, the impact on biological activity, which can be split into two distinct modes of action, antigen binding and Fc function, was independently assessed for each mAb.

### Binding to cell-surface antigens is increased after Trx reduction for rituximab, ofatumumab, and cetuximab but not for trastuzumab

**Figure 5.**
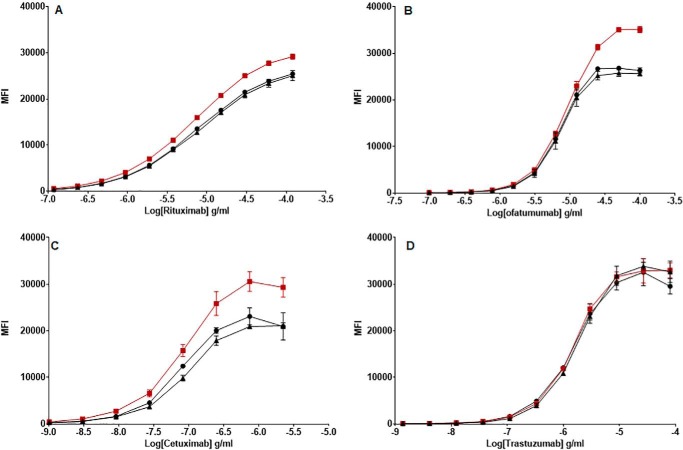
**Antigen binding of Trx-reduced mAbs.** Target antigen–expressing cells CD20 Wil2-S cells for rituximab (*A*) and ofatumumab (*B*), EGFR HER1 A431 cells for cetuximab (*C*), and HER2 BT474 for trastuzumab (*D*) were incubated with Trx-treated (■), NEM-only-treated (▴), and untreated (●) control mAbs at different concentrations. Bound mAbs were detected using PE-conjugated anti-human IgG1 via flow cytometry. The data were gated and analyzed using FlowJo software with respect to a shift in PE median MFI. For all experiments, nonspecific isotype controls were included; trastuzumab (used for *A* and *B*) and rituximab (used for *C* and *D*) generated values comparable with unstained controls (not shown). MFI values were used to generate dose-response curves. The data shown is representative of data from at least three individual experiments. *Error bars* (present although not observable for all points), S.D. of replicates; *n* = 3.

Four of the antibodies (rituximab, ofatumumab, cetuximab, and trastuzumab) primarily bind to antigens expressed on the surface of target cells; therefore, we examined the antigen-binding capacity of Trx-reduced mAbs to their target cells. After incubation of Trx-treated and control mAbs with target cells, binding was determined using a fluorescently labeled secondary antibody. Median fluorescence intensity (MFI) values were derived from flow cytometric analysis of mAb-bound target cells, and dose-response curves were generated. Binding of all four mAbs to their target cells generated sigmoid dose-response curves (Fig. 5; representative raw flow cytometry data are shown in Fig. S2). Trx reduction impacted the antigen binding of rituximab, ofatumumab, and cetuximab, where curves were shifted slightly, showing higher maximal MFI values compared with the untreated and *N*-ethylmaleimide (NEM)-treated controls. In contrast, antigen binding by trastuzumab was unaffected by Trx treatment. In general, treatment with NEM did not affect the shape of the dose-response curves. The relative binding affinity of Trx-treated mAbs compared with their cognate nonreduced controls was determined by the ratio of the calculated EC_50_ values. For each mAb, the geometric mean (GM) of this ratio was determined, with lower and upper 95% confidence limits (LCL and UCL, respectively) from three independent experiments to ensure the robustness of the findings (Table 3). The observed variability across the assays was low, with geometric coefficient of variation (GCV) ranging from 3 to 11%. For NEM treatment alone, there was no significant difference in EC_50_ ratios, confirming that NEM treatment had a minimal impact upon antigen binding. For rituximab (Fig. 5*A*), ofatumumab (Fig. 5*B*), and cetuximab (Fig. 5*C*), Trx reduction resulted in increased EC_50_ ratios (1.56, 1.52, and 2.05, respectively), implying increased affinity or avidity of the antibody following reduction, whereas the ratio for trastuzumab (Fig. 5*D*) was 0.96. One possible explanation for the apparent increase in binding of the mAbs to their cell-surface antigens could be due to the secondary fluorescent antibodies binding differentially to the control and Trx-reduced therapeutic mAbs, respectively. To explore this possibility, three mAbs (infliximab, where no cell-surface binding was explored; cetuximab, where Trx reduction enhanced cell-surface binding; and trastuzumab, where Trx reduction had no effect upon cell-surface binding) were titrated and directly bound to ELISA plates in both their native and Trx-reduced forms. Horseradish peroxidase–conjugated anti-κ and anti-Fc antibodies were then bound to the therapeutic mAbs and detected with a 3,3′,5,5′-tetramethylbenzidine substrate. The resultant binding curves are shown in Fig. S3, and analysis of the EC_50_ ratios of anti-κ and anti-Fc binding to Trx-reduced mAbs relative to nonreduced controls shows that the binding levels are all very similar (within 10%; Table S1). As this binding of the secondary antibodies to the control and Trx-modified mAbs is independent of antigen, any differences in mAbs binding to cell-surface antigens is due to modulation of their interaction due to Trx reduction and not due to different levels of secondary antibody binding. Therefore, disulfide bond reduction of mAbs either enhances or has no impact on target cell-surface antigen interactions.

**Table 3:** **Assessment of antigen binding by Trx-treated and NEM-treated control mAbs** GM of potency estimates with LCL and UCL were calculated using EC_50_ ratios of NEM-treated or Trx-treated mAbs to untreated control from three independent experiments. Homogeneity (*p* > 0.1) was assessed using a weighted combination of potency estimates. GCV was calculated from individual GM values (not shown) as a measure of assay variability.

	Trx-reduced *versus* untreated control EC_50_ ratios				NEM-treated control *versus* untreated control EC_50_ ratios			
	Rituximab	Ofatumumab	Cetuximab	Trastuzumab	Rituximab	Ofatumumab	Cetuximab	Trastuzumab
GM	1.56	1.52	2.05	1.07	0.98	0.95	0.95	0.93
GCV (%)	3.50	3.20	10.80	10.80	4.28	2.76	4.25	2.79
LCL	1.52	1.49	1.88	0.96	0.95	0.92	0.92	0.84
UCL	1.61	1.55	2.23	1.21	1.02	0.97	0.99	1.03

### The TNF neutralization potency of infliximab and adalimumab is increased after reduction by Trx

**Figure 6.**
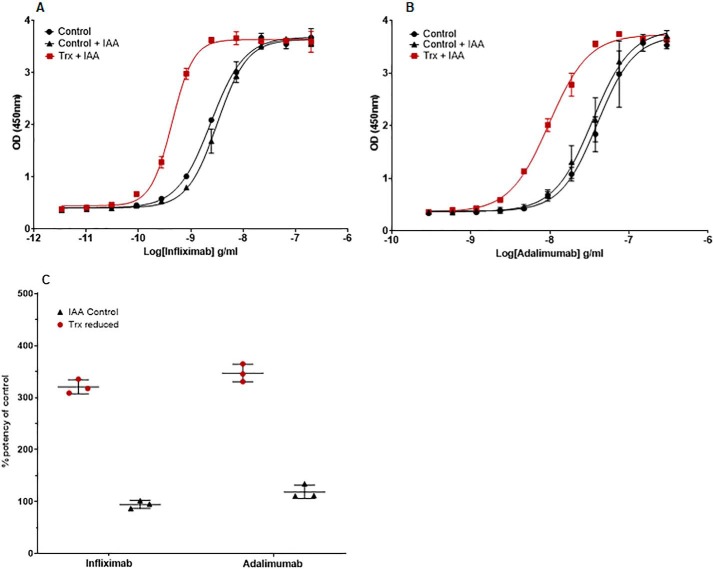
**Bioactivity of Trx reduced anti-TNF mAbs.** Both infliximab (*A*) and adalimumab (*B*) were reduced with the Trx system, and the neutralizing activity of both mAbs was determined by assessing the cytotoxic effect of TNF on WEHI-164 cells after 18 h. Cell viability was measured using the water-soluble formazan dye CCK-8 by absorbance at 450 nm for Trx-treated (■), NEM-only-treated (▴), and untreated control (●) mAbs. ED_50_ values were calculated by fitting a four-parameter sigmoidal curve to the data. The data shown is representative of data from one assay of at least three individual experiments. *Error bars* (present although not observable for all points), S.D. of replicates; *n* = 2. *C*, mean potencies and S.D. of three experiments relative to the nonreduced control sample are shown for Trx-reduced and IAA-treated infliximab and adalimumab. Each experiment consisted of two assay plates from which the relative potencies were averaged.

TNF neutralization is the main therapeutic mechanism of action of the two anti-TNF mAbs, infliximab and adalimumab, and is mediated via the binding of their Fab regions to TNF. The potency of Trx-reduced infliximab and adalimumab relative to untreated and IAA-treated controls were determined in TNF neutralization bioassays employing the TNF-sensitive mouse fibrosarcoma WEHI-164 cell line. Dose-dependent neutralization of the cytotoxic effect of TNF was observed for all of the infliximab (Fig. 6*A*) and adalimumab (Fig. 6*B*) samples. EC_50_ values were calculated for each sample, and relative potencies of the samples were calculated from ratios of the EC_50_ values (Fig. 6*C*). The potencies of IAA-treated samples were, as expected, similar to the untreated samples for both infliximab and adalimumab: 95 and 119%, respectively (potencies of between 70 and 130% are generally considered to be acceptable in bioassays). However, after Trx reduction, the potency of both infliximab (320% with a CV of 4.3%) and adalimumab (347% with a CV of 4.9%) increased significantly relative to untreated controls. Again, this suggests that antibody-antigen binding is enhanced by Trx reduction.

### The antiproliferative activity of trastuzumab is reduced after Trx reduction

**Figure 7.**
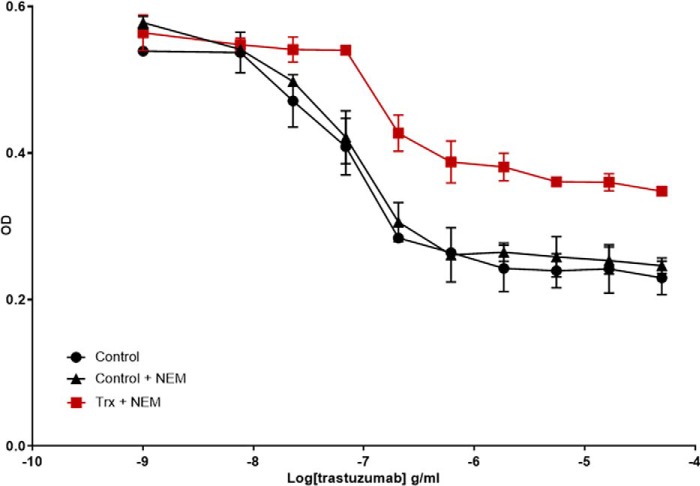
**Assessment of Trx reduced trastuzumab antiproliferative activity.** The antiproliferative activity of control trastuzumab (●), Trx-treated trastuzumab (■), and NEM-treated control trastuzumab (▴) was determined by assessing the growth of HER2-expressing BT474 cells by using the alamarBlue viability dye and measuring the absorbance (570 nm). The data shown is representative of data from at least three individual experiments. *Error bars* (present although not observable for all points), S.D. of replicates; *n* = 3.

The antigen-binding capacity of the mAbs increases following Trx reduction except for trastuzumab, whose ligand binding is unchanged (Fig. 5*D*). The antiproliferative effect of trastuzumab is largely determined by HER2 antigen binding; we examined this *in vitro* using the HER2-expressing BT474 cell line. A dilution series of Trx-reduced trastuzumab was incubated with BT474 cells (or untreated or NEM-treated controls) for 5 days, after which proliferation was assessed indirectly using alamarBlue (Fig. 7). Dose-dependent growth inhibition was observed with increasing concentrations of untreated and NEM-treated controls, whereas the antiproliferative activity decreased when trastuzumab was reduced with Trx reduction. These data suggest that, in contrast to the anti-TNF mAbs, trastuzumab potency is decreased after Trx reduction.

### Trx reduction abolishes the ability of therapeutic mAbs to interact with and stimulate the immune system

**Figure 8.**
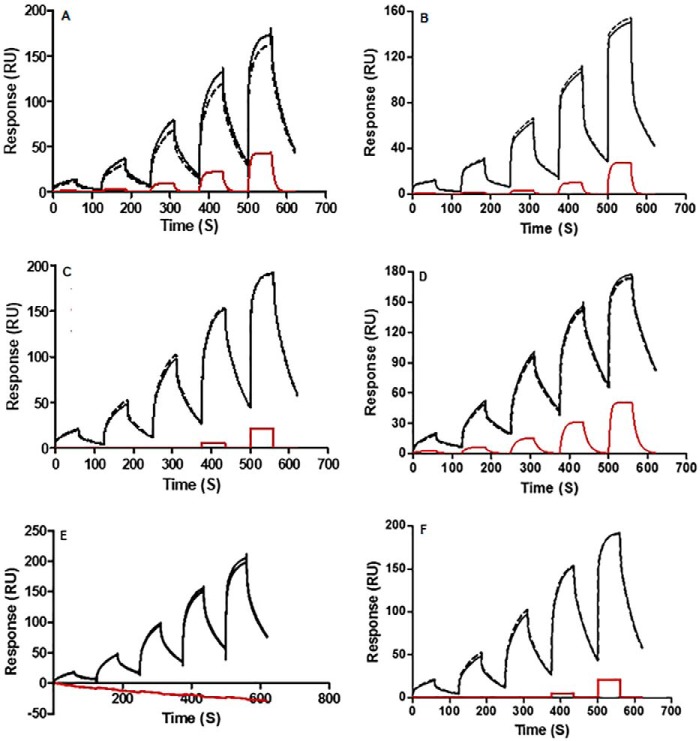
**SPR was performed for mAbs to assess binding to the ADCC-relevant receptor CD16.** Five injections of untreated control (*solid black line*), NEM-only-treated control (*dashed black line*), and Trx-treated (*solid red line*) mAbs (25–2025 nm) were loaded in increasing concentrations across surface-bound CD16 as detailed under “Experimental procedures” for rituximab (*A*), ofatumumab (*B*), cetuximab (*C*), trastuzumab (*D*), infliximab (*E*), and adalimumab (*F*). Sensorgrams were generated using two-state reaction analysis of experimental data with Biacore (T100) evaluation software.

**Figure 9.**
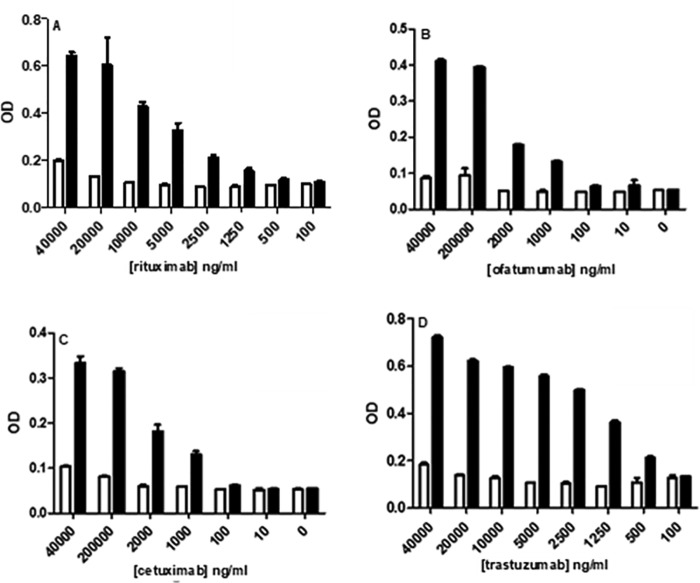
**Binding of Trx-treated (*white*) and untreated control (*black*) mAbs to plate-bound CD16 was detected by ELISA as described for rituximab (*A*), ofatumumab (*B*), cetuximab (*C*), and trastuzumab (*D*).** OD readings measured at 450 nm correlated with binding. The data shown is representative of data from at least three individual experiments. *Error bars* (present although not observable for all points), S.D. of replicates; *n* = 3.

mAbs engage the immune system via their Fc regions, which interact with Fc receptors on the surface of immune cells. It is well-established that structural modifications to mAbs can alter their Fc-mediated immunobiological activities. For example, the absence of fucose within the *N*-linked glycan present on the Fc is well-documented to enhance antibody-dependent cellular cytotoxicity (ADCC) activity (33, 34). In particular, the affinity and stability of the interaction of mAbs with the FcγRIII (CD16) receptor has been reported to strongly influence the activation of natural killer (NK) cells (35). To probe the biochemistry of this interaction, we employed both surface plasmon resonance (SPR) single-cycle kinetics and solid-state ELISA assays. In SPR assays, Trx-treated mAbs demonstrated a decrease in their interaction with surface-bound CD16 compared with untreated mAbs (Fig. 8). Furthermore, Trx-treated cetuximab showed no evidence of binding. These data were further supported in ELISAs, in which binding to immobilized CD16 was also clearly inhibited following treatment of all of the antibodies with Trx (Fig. 9). Thus, interchain disulfide bond reduction by Trx disrupts binding of the Fc region to CD16.

**Figure 10.**
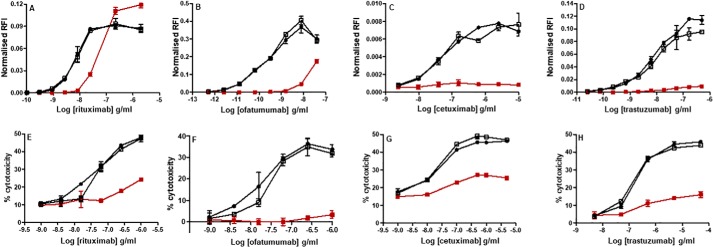
**ADCC activity of Trx-reduced mAbs.** Activity of Trx-treated mAb (■), NEM-only-treated mAb (□), and untreated control (●) were assessed using orthogonal ADCC assay readouts using target cells against either CD16-expressing surrogate or LAK effector cells. ADCC activity of rituximab (*A*), ofatumumab (*B*), cetuximab (*C*), and trastuzumab (*D*) was measured using the J9 reporter gene cell line with appropriate target cells. Dose-response curves were generated using normalized luciferase expression as described under “Experimental procedures.” Note that the *y* axis scales are not equivalent and are indicative of day-to-day fluctuation and/or sensitivity of the assay with the target cells. ADCC activity of rituximab (*E*), ofatumumab (*F*), cetuximab (*G*), and trastuzumab (*H*) was measured using IL-2–activated LAK cells against calcein AM–loaded target cells. Target cell cytotoxicity (subtracted from background) was calculated as described under “Experimental procedures.” The data shown is representative of data from at least three individual experiments. *Error bars* (present although not observable for all points), S.D. of replicates; *n* = 3.

Having discovered that Trx-mediated mAb reduction impairs binding to CD16, we next investigated whether this translates to an abrogation of the immune response. As a key mechanism of action common to all of the mAbs, the effect of Trx reduction on ADCC activity was assessed using several bioassays. We have reported previously that the J9 (CD16-expressing) cell line–based luciferase reporter gene assay is a specific and sensitive surrogate method for the measurement of ADCC activity (36). Dose-response curves for Trx-reduced mAbs, along with untreated and NEM-treated controls, were constructed (Fig. 10), and EC_50_ ratios were determined. Ratios of EC_50_ values show that for all of the mAbs, NEM treatment alone did not impact their ADCC activity (Table 4), whereas for both Trx-treated anti-CD20 mAbs, luciferase activity was decreased, indicating impaired ADCC activity. It is interesting to note that rituximab (Fig. 10*A*) retained more activity than ofatumumab (Fig. 10*B*). Neither Trx-treated cetuximab (Fig. 10*C*) nor trastuzumab (Fig 10*D*) was unable to induce significant luciferase gene expression, indicating a complete abrogation of ADCC activity. Our previous characterization of the J9 cell line had revealed that suspension cells demonstrate greater sensitivity as targets than adherent cells (36). As the target cells for cetuximab and trastuzumab are adherent, this may explain why they show less ADCC activity compared with rituximab and ofatumumab, which are directed against suspension target cells.

**Table 4:** **Assessment of NEM-treated and untreated control J9 ADCC activity** GMs of potency estimates with LCL and UCL were calculated using EC_50_ ratios of NEM-treated/untreated control mAbs from three independent experiments. Homogeneity (*p* > 0.1) was assessed using a weighted combination of potency estimates. GCV was calculated from individual GM values (not shown) as a measure of assay variability.

	Trx-reduced *versus* untreated control EC_50_ ratios				NEM-treated control *versus* untreated control EC_50_ ratios			
	Rituximab	Ofatumumab	Cetuximab	Trastuzumab	Rituximab	Ofatumumab	Cetuximab	Trastuzumab
GM					1.0	1.0	1.0	0.9
GCV (%)					11.5	0.9	6.6	14.1
LCL					1.0	0.9	0.9	0.8
UCL					1.1	1.1	1.0	1.1

The J9 reporter gene assay is highly sensitive and can resolve small differences in ADCC activity, but it does not measure cell killing *per se*. Therefore, to address this, the reduced mAbs were assayed using *ex vivo* lymphokine-activated killer (LAK) cells. LAK cells were purified from human leukocyte cones and activated with IL-2. As with the J9 surrogate assay, a sigmoidal dose-response curve was established using a similar range of mAb concentrations for rituximab (Fig. 10*E*), ofatumumab (Fig 10*F*), and cetuximab (Fig. 10*G*). For trastuzumab (Fig. 10*H*), a 100-fold higher concentration was required to see a cytotoxic effect (500 ng/ml *versus* 50 μg/ml). All NEM control samples showed ADCC activity comparable with that of the untreated control samples, but reduction by Trx largely abrogated the ADCC activity of all mAbs. It was not possible to assess the ADCC activity of infliximab and adalimumab, as we did not have access to a suitable mTNF target cell line.

**Figure 11.**
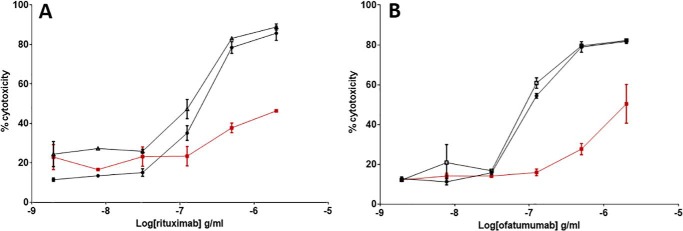
**CDC activity of Trx-treated mAbs.** Untreated control (●), Trx-treated (■), and NEM-only-treated control (□) rituximab (*A*) and ofatumumab (*B*) were incubated with calcein AM–loaded Wil2-S target cells, after which rabbit complement was added. Target cell cytotoxicity was calculated as described. The data shown is representative of data from three individual experiments. *Error bars* (present although not observable for all points), S.D. of replicates; *n* = 3.

Another important Fc-mediated mechanism of immune activation is complement-dependent cytotoxicity (CDC), which was investigated for both anti-CD20 mAbs rituximab and ofatumumab. CD20-expressing Wil2-S target cells were incubated with the mAbs in the presence of rabbit complement. Sigmoidal dose-dependent cytotoxicity was observed for both untreated mAb and NEM-treated controls, both of which achieved similar maximal levels of cytotoxicity over identical dose ranges for both rituximab (Fig. 11*A*) and ofatumumab (Fig. 11*B*). However, as seen for ADCC, CDC-mediated cytotoxicity was abrogated after Trx treatment.

**Figure 12.**
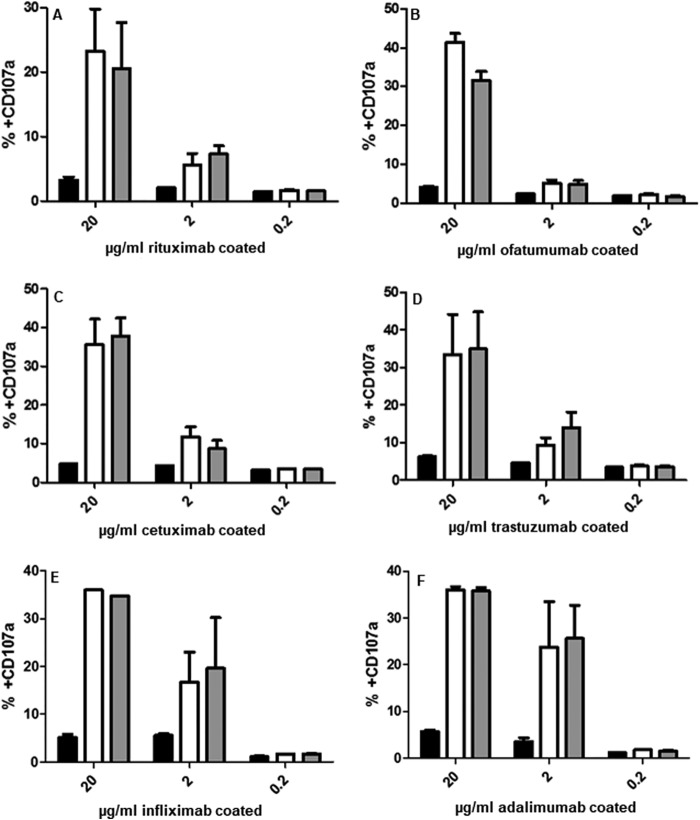
**Degranulation of NK cells after incubation with plate-bound dose responses of rituximab (*A*), ofatumumab (*B*), cetuximab (*C*), trastuzumab (*D*), infliximab (*E*), and adalimumab (*F*) was examined using flow cytometry, and the percentage of the cell population that was CD107a^+^ was determined.** Trx-treated (*black*), NEM-only control (*gray*), and untreated control mAbs (*white*) were assessed. The data shown is representative of data from at least three individual experiments. *Error bars* (present although not observable for all points), S.D. of replicates; *n* = 3.

Thus far, it is clear that Trx reduction of the mAbs abrogates their ADCC and CDC activity. However, these assays also rely on the binding of mAbs to their antigens on target cells, a process that, as discussed earlier, is also modulated by Trx reduction (Figs. 5 and 6). To isolate the Fc function of the modified mAbs, the activation of LAK cells by plate-bound mAbs was assayed with respect to the induced expression of CD107a, a marker of degranulation. Following incubation with immobilized mAbs, CD107a-positive LAK cell populations were determined by flow cytometry (Fig. 12). As expected, both untreated and NEM-treated controls were able to mediate marked LAK cell activation, as observed at the highest dose, 20 μg/ml for all of the mAbs (including infliximab (Fig. 12*E*) and adalimumab (Fig. 12*F*)). In contrast, Trx-treated mAbs were unable to activate LAK cells and induce degranulation, even at the highest concentration, where only a marginal increase was observed. Combined, these data suggest that the abrogation of mAb Fc-mediated activity is due to modulation of their interaction with immune Fc receptors and is independent of any modulation in antigen-binding activity.

### Trx reduction of mAb interchain disulfide bonds is reversible, resulting in restored biological activity

**Figure 13.**
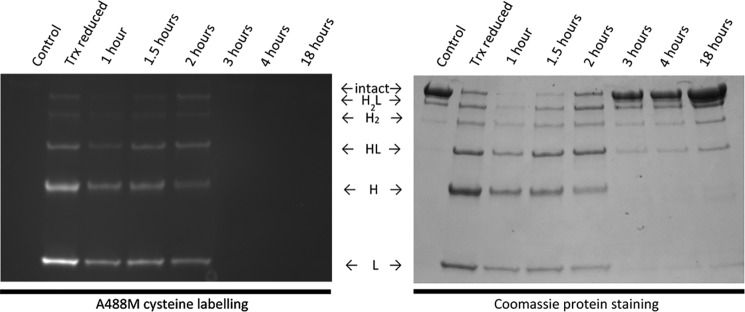
**Reoxidation of Trx reduced rituximab in the absence of alkylating agent.** Nonreducing SDS-PAGE of rituximab shows the time-dependent incorporation of the cysteine label A488M after reduction with Trx. Gels were visualized at 488 nm to probe A488M cysteine labeling (*left*) and Coomassie-stained for total protein (*right*).

In the experiments carried out thus far, the interchain thiol groups liberated from Trx reduction of disulfide bonds have been prevented from reoxidation by alkylation with either IAA or NEM. This has allowed evaluation of both the physicochemical properties of Trx-reduced mAbs and the effect on biological activity. It has been known for some time that reduced disulfide bonds can reform, and this has been well-documented in proteins such as interferon-γ, insulin, and Igs (37). However, most of this work has been carried out using chemical reductants such as DTT and tris(2-carboxyethyl)phosphine (TCEP), and the mAbs have not been subjected to the protein folding and chaperoning functions of Trx (38). To evaluate the reoxidation of disulfide bonds within the mAbs after reduction by Trx, A488M was added to mAbs at different time points after Trx treatment. For all mAbs (Fig. 13; rituximab is shown), fluorescent A488M-labeled protein bands corresponding to heavy and light chain bands or combinations thereof were present after 90 min in the presence of Trx at 37 °C. These bands were diminished after 3 h (A488M cysteine labeling) with a concomitant increase in the appearance of a 150 kDa band (Coomassie staining) corresponding to reformation of the fully intact mAb. This was the dominant species at 18 h, confirming that reduction of interchain disulfide bonds by Trx is reversible.

**Figure 14.**
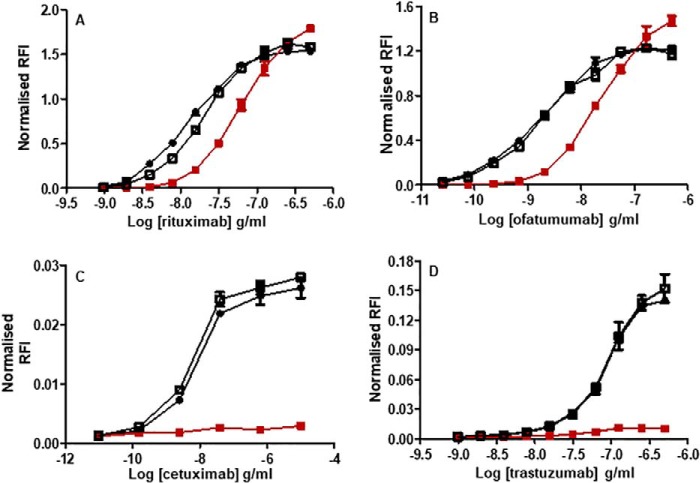
**ADCC activity of reoxidized mAbs.** Trx-reduced mAbs, confirmed by SDS-PAGE, were allowed to reoxidize over time, and their ADCC activity was measured using a J9 reporter gene cell line–based assay with respective target cells. Activities of reoxidized mAbs (□) were assessed alongside untreated control (●) and Trx-reduced mAbs (■). Dose-response curves were generated using normalized luciferase expression as described. Note that *y* axis scales are not equivalent and are indicative of day-to-day fluctuation and/or sensitivity of the assay with target cells. The data shown is representative of data from at least three individual experiments. *Error bars* (present although not observable for all points), S.D. of replicates; *n* = 3.

To investigate whether the disulfide bonds have reformed in a physiologically relevant manner and hence restored Fc effector activity, the reoxidized mAbs were subjected to surrogate ADCC assays. The ADCC activity for all reoxidized mAbs compared with their respective untreated controls was highly similar (Fig. 14). The EC_50_ ratios determined for reoxidized trastuzumab and ofatumumab compared with the untreated controls were 1.0 and 1.1, respectively, showing a complete recovery of activity. The potency of reoxidized rituximab was slightly lower (estimated to be 0.87), possibly due to incomplete reoxidation. Overall, this confirmed that Trx reduction of mAb disulfide bonds is reversible and that biological activity is mostly restored when the bonds reform.

## Discussion

Gaining an understanding of the functional consequences of post-translational modifications of therapeutic mAbs is important, as unplanned or unwanted modifications can impact their safety and efficacy. Post-translational modification of labile disulfide bonds by Trx is a well-established molecular mechanism whereby cells modulate the structure and function of their proteins. The work presented herein highlights the *in vitro* biological impact of Trx-mediated reduction on six therapeutic mAbs: infliximab, adalimumab, rituximab, trastuzumab, ofatumumab, and cetuximab. All mAbs are administered in cancer or inflammatory disease states where high levels of Trx have been reported. Recently, tregalizumab, a mAb targeting CD4 on regulatory T cells in RA, failed clinical trials because the high levels of Trx in RA patients reduced a disulfide bond in CD4, which in turn decreased the activity of tregalizumab (29), so it is not without precedent that Trx or other oxidoreductase enzymes could reduce some of the many disulfide bonds in therapeutic mAbs.

The function of mAbs can be broadly divided into two distinct mechanisms: antigen binding and Fc-mediated functions. Here, we report that these two functions can be independently modulated by Trx.

Although Trx is an efficient reducer of disulfide bonds, not all disulfide bonds in a protein are substrates for Trx (39). The reactivity of disulfide bonds depends upon a number of factors, including how accessible they are to the enzyme (surface-exposed bonds are more reactive) or their conformation (40). All of the mAbs in this study are of the IgG1 subclass, which is the most common class of therapeutic mAbs (41). Infliximab, rituximab, and cetuximab are chimeric mouse/human, trastuzumab is humanized, and adalimumab and ofatumumab are fully human mAbs. X-ray crystallography shows that interchain bonds present in the flexible hinge region of IgG1 are 25–50% exposed to the surroundings (42) whereas intrachain bonds are hidden within the Ig domains, specifically between two layers of β sheet (43). Mass spectrometry–based analysis revealed that under native conditions, intrachain bonds are inaccessible to chemical reducing agents, whereas interchain bonds are susceptible to chemical reduction. In contrast to chemical reductants, the more physiological oxidoreductase enzyme Trx has the ability to partially unfold certain Ig domains and reduce disulfide bonds at their core (12). However, when we analyzed Trx-treated infliximab, rituximab, and trastuzumab by various analytical methods, they all showed no intrachain disulfide bond reduction, and only the interchain bonds were reduced. This confirms that the Ig domains of the mAbs are not unfolded by Trx, and they remain largely conformationally intact. This reduction *per se* only induces minor structural perturbations.

Previous studies have shown that the affinity of trastuzumab for its target antigen were unchanged following chemical (DTT) reduction and subsequent alkylation, whereas for rituximab, they were slightly increased (44, 45). Here, using more physiologically relevant enzymatic Trx reduction, we see a similar trend of increased antigen binding for rituximab and no change in antigen binding for trastuzumab, again indicating that the same disulfide bonds are reduced by DTT and Trx. Additionally, for ofatumumab and cetuximab, we see an increase in antigen binding, but no effect on antigen binding for the anti-TNF mAbs infliximab and adalimumab. Once a mAb binds to its target antigen, it can have a direct functional effect (*e.g.* neutralizing a soluble antigen, preventing it from binding to a receptor; blocking a receptor from binding; or disrupting or promoting cross-linking of cell-surface molecules). Fc-mediated mAb function, such as ADCC or CDC, results in the killing of the target cell, and we have shown here that for all of the mAbs where assays were available (rituximab, ofatumumab, cetuximab, and trastuzumab), ADCC activity is diminished after Trx reduction. We determined that this was the because their binding to CD16, the activating FcγIIIa receptor, was abolished after Trx reduction. In addition, we found a loss of degranulation in cytotoxic cells for all mAbs tested (including infliximab and adalimumab, where no ADCC assay was available) after Trx reduction. As degranulation is independent of antigen binding, this shows that the effect of Trx on Fc function overrides the increase in antigen binding and reveals that the two functions of therapeutic mAbs are independently modulated following Trx reduction. This is further highlighted for infliximab and adalimumab, where, remarkably, and in contrast to their Fc activity, neutralization of the pro-inflammatory cytokine TNF is increased severalfold after treatment with Trx.

It is clear from data shown here and from previous studies that reduction of interchain disulfide bonds in IgG1 antibodies can dramatically alter their function. Undoubtedly, this is mediated by a change in the flexibility of the molecule as a whole due to decreased rigidity of the hinge region upon reduction of the two HC-HC disulfide bonds. Even with these bonds intact, IgG1 is the second most flexible subclass of IgG (46), which was further highlighted when the first crystal structure of a human HIV-1–neutralizing IgG1 mAb (b12) was obtained (47). The structure of b12 shows significant asymmetry, deviating considerably from the classical “Y-shaped” arrangement of Fab and Fc regions to an almost “T-shaped” arrangement. This flexibility allows the long finger-like CDR H3 to penetrate deep inside a cleft on the GP-120 antigen. Interestingly, one of the two disulfide bonds in the hinge region of the structure is reduced, probably by radiation damage, but the authors conclude that this does not impact the flexibility of the region. However, when too much flexibility is introduced into the hinge region, Fc-mediated activity is compromised. A rational mutational study on the mAb 12G3H11, which binds to human EphA2, showed that any mutations introducing further flexibility into the hinge region, along with mutation of each of the disulfide bonds in turn, had a detrimental effect on CD16 and C1q complement binding, resulting in reduced CDC and ADCC activity (48). Therefore, it is reasonable to conclude that reduction of both HC-HC disulfide bonds in IgG1 mAbs by Trx results in increased flexibility.

The mechanisms by which this increased flexibility leads to inhibition of Fc function are probably a combination of two independent contributions. First, increased flexibility allows the Fab domains to become more mobile and therefore increases their potential to sterically inhibit the interaction between the Fc receptors and the Fc domain of the mAb interaction. Second, this interaction is extremely sensitive to structural perturbations; for example, methionine oxidation in the Fc domains of two mAbs causes structural perturbations at the CH2-CH3 domain interface, resulting in decreased Fc effector function (49, 50). Any similar structural perturbations in the CH2 and CH3 domains imparted by the Trx reduction of the HC-HC disulfide bonds could also impact Fc effector function directly.

In direct contrast to these observations, increased flexibility in the hinge region upon Trx reduction could easily account for the remarkable increase in TNF neutralization potency for infliximab and adalimumab. TNF is trimeric in solution, and crystallographic studies show that each TNF trimer can bind three mAb Fab fragments (51, 52). As a result, with full-length bivalent mAbs, large immune complexes can form between infliximab or adalimumab and trimeric TNF (53, 54). However, the more rigid the mAb structure, the greater role that steric hindrance plays in restricting the size of these complexes. It is likely, therefore, that more flexible Trx-reduced mAbs could form larger complexes, effectively increasing the number of TNF molecules neutralized per molecule of mAb, thus effectively increasing the TNF neutralization potency.

The decrease in antiproliferative activity of Trx-reduced trastuzumab on BT474 can similarly be explained by an increase in flexibility. HER2 overexpressed on the surface of the cells results in receptor clustering, constituent signaling, and continued proliferation. Trastuzumab disrupts the HER2 clustering, thus decreasing the proliferation signal to the cells (55, 56). However, a loss of rigidity in trastuzumab could reduce its effectiveness in inhibiting HER2 interactions and clustering, thus diminishing antiproliferative effects.

In conclusion, *in vitro* Trx reduction of the labile interchain disulfide bonds in therapeutic mAbs has a profound effect on their function. The actual functional effects are dependent upon the mode of action of each mAb, but in each case, they adopt functionality that is very different from the unmodified mAb. As high levels of Trx are found in many autoimmune and cancer disease states, these therapeutic mAbs will be exposed to these conditions and likely modified in a way similar to the changes described here. Moreover, mAbs will be far less stable in the harsher extracellular environment and more susceptible to aggregation, proteolysis, and degradation. Thus, not only will the therapeutic potential of the mAbs be directly impacted, but they could become more immunogenic. However, detection of these modified mAbs *in vivo* will present challenges, as Trx reduction is likely to take place locally at sites of inflammation, meaning that the majority of mAb in the peripheral circulation will be in the native state. Furthermore, we have shown that, without freezing the redox state with alkylating agents, the effects of Trx reduction are reversible, making detection even harder.

This study lays the foundations of determining whether *in vivo* Trx reduction impacts the safety and efficacy of therapeutic mAbs, paving the way for future development of safer, more effective biological therapies and treatment regimes.

## Experimental procedures

### Cell culture

Human B cell lines WIL2-S (lymphoblastoid), Raji (derived from a Burkitt's lymphoma patient), and Daudi (lymphoblastoid) (all NIBSC stock) were utilized between passages 3 and 30 and cultured in complete medium, consisting of Roswell Park Memorial Institute (RPMI) 1640 (Sigma-Aldrich) containing 10% fetal calf serum (FCS; Labtech; South American origin) and penicillin/streptomycin (50 IU/50 ng/ml) and 2 mm glutamine (both from Sigma-Aldrich). Medium was refreshed every 3–4 days. For Daudi cells, the medium was supplemented with 1 mm sodium pyruvate (Sigma-Aldrich). A431 epidermal cells (NIBSC stock) were cultured in Dulbecco's modified Eagle's medium (DMEM; Sigma-Aldrich) containing 10% FCS, penicillin/streptomycin (50 IU/50 ng/ml), and 1% glutamine. BT474 human breast cancer cells (ATCC, Middlesex, UK) were cultured in DMEM/F-12 medium (Sigma-Aldrich) containing 10% FCS and penicillin/streptomycin (50 IU/50 ng/ml)-supplemented and used between passages 4 and 20. WEHI-164 cells (CRL-1751, ATCC) were cultured in RPMI 1640 (30-2001, ATCC), 10% FCS-supplemented, and used between passages 2 and 10. For both BT474 and WEHI-164 cells, the medium was refreshed every 3 days; after a 4-day period, cells were trypsinized, and the density was adjusted to 1 × 10^5^ cells/ml. The transfected J9 cell line (ENS Cachan, Villejuif, France) was cultured in RPMI complete medium (as detailed above) supplemented with 150 μg/ml hygromycin, 50 μg/ml zeocin, and 12.5 mm puromycin (InvivoGen) between passages 3 and 15.

Peripheral blood mononuclear cells were isolated from leukoreduction system chambers, denoted as “cones” (obtained from the NHS Blood and Transplant Service, Colindale, UK) within 18–24 h of collection. The volume contained in cones (∼10 ml) was diluted with 50 ml RPMI prior to separation using Histopaque-10771 (Sigma-Aldrich). Following negative selection of NK cells with a human NK cell isolation kit (Miltenyi Biotec, Bergisch Galdbach, Germany), the cells, which after expansion are referred to as LAK cells, were cultured in LAK cell culture medium consisting of 55% DMEM (Sigma-Aldrich), 30% F-12, 1× nonessential amino acids, 1 mm sodium pyruvate, and 50 μm mercaptoethanol (Gibco), 0.1 mg/ml penicillin/streptomycin with IL-2 at 200 IU/ml (Centre for AIDS Reagents, NIBSC). Medium, including IL-2, was refreshed every 6 days.

### Reagents

Anti-CD20 antibody rituximab, denoted as rituximab (MabThera), and anti-HER2 trastuzumab (Herceptin) (both from Roche (Basel, Switzerland)), anti-HER1 cetuximab (Erbitux, Merck, Darmstadt, Germany), anti-CD20 ofatumumab (Arzerra, Novartis (Basel, Switzerland)), and anti-TNF mAbs adalimumab (Humira, AbbVie) and infliximab (Remicade, Johnson and Johnson) are therapeutic products. Other reagents used were phycoerythrin (PE)-conjugated anti-human IgG (Fcγ-specific), anti-human Brilliant Violet-421 anti-human CD107a (Cambridge Bioscience, Cambridge, UK), and monensin solution (GolgiStop, BD Biosciences).

### Thioredoxin or tris(2-carboxyethyl)phosphine reduction

Reactions were carried out using 10-kDa cut-off spin filters (Sartorius, Cologne, Germany), referred to as filter-aided sample preparation (FASP). PBS (200 μl) containing 5 mm TCEP or 1 μm thioredoxin with 33 mm NADPH and 680 nm thioredoxin reductase (Sigma-Aldrich) (premixed for 10 min at RT) were added per 500 μg of mAb for 90 min at 37 °C. From this reaction, 62.5 μg was removed, placed onto a new filter, and quenched with Alexa-488-maleimide (Thermo Fisher Scientific, Hemel Hempstead, UK). The remaining reaction was quenched with 50 mm NEM (Sigma-Aldrich) or IAA and incubated for 1 h in the dark at RT. mAbs were washed with PBS using, and reconstituted in, PBS to ∼2 mg/ml, confirmed by NanoDrop (Thermo Fisher Scientific). NEM or IAA controls were carried out and processed in parallel.

### SDS-PAGE

Ten μg mAb in nonreducing Laemmli sample buffer (Bio-Rad) was incubated at 95 °C for 10 min before running on Mini-PROTEAN TGX gels (Bio-Rad) at 150 V for 1 h. Coomassie staining or fluorescence was visualized with a GeneSnap gel imager (Syngene, Cambridge, UK) using visible protein or Alexa-488 filter protocols, respectively.

### Kinetic trapping of reduced disulfide bonds in mAbs and preparation for MS analysis

Each mAb (50 μg in PBS of infliximab, rituximab, or trastuzumab) was added to a 10-kDa 500-μl centrifugal concentrator (Vivacon 500, Sartorious) and reduced with Trx (100 μl of 1 μm Trx supplemented with 100 nm TR1 and 200 μm NADPH for 90 min at 37 °C). After washing with 500 μl of PBS, free cysteines arising from the reduction of disulfide bonds were alkylated with IAA (100 μl of 50 mm in 25 mm ammonium bicarbonate for 1 h at RT in the dark). A non-Trx-reduced control sample was treated with IAA only to obtain the base level of free cysteines in the mAbs. All samples were denatured, and any remaining disulfide bonds were chemically reduced (200 μl of 8 m urea solution with 25 mm TCEP for 1 h at RT), after which the tubes were centrifuged to remove liquid from filters. Cysteines arising from this were alkylated with NEM (200 μl of 50 mm in PBS for 60 min at room temperature) to discriminate them from cysteines arising from Trx reduction. A 100% IAA-labeled sample of each mAb was also prepared by denaturing and reducing 50 μg of antibody in a centrifugal concentrator and alkylating with IAA as above. Three biological replicates were prepared for each mAb. The samples were trypsin-digested, as reported previously (12). For this, samples were then incubated with 2 μg of trypsin (Sigma) in 100 μl of Ambic at 37 °C overnight. Any remaining liquid was then spun into a clean collection tube, and 100 μl of 0.1% formic acid was added to each sample for 10 min before centrifuging the liquid into the collection tube. Solutions of 50 and 100% acetonitrile with 0.1% formic acid were then consecutively added to the samples, which were centrifuged to remove liquid at each stage. The filters were then removed, and samples were placed in a SpeedVac (Thermo Fisher Scientific) at 45 °C to remove all liquid. For MS, samples were reconstituted in 0.1% formic acid, followed by sonication.

### Mass spectrometry

The tryptic peptide samples were injected as technical triplicates onto an Ultimate 3000 nano-HPLC system coupled to an Orbitrap XL Discovery mass spectrometer (Thermo Scientific). Samples were online desalted on a μ-Precolumn (C18 PepMap100, 300-μm inner diameter × 5 mm; 5 μm, 100 Å) at a flow rate of 25 μl/min, which was followed by separation on a nano-analytical column (Acclaim PepMap100 C18, 75-μm inner diameter × 50 cm, 3 μm, 100 Å) (Thermo Scientific) using a 90-min linear gradient from 5 to 40% solvent B (98% CH_3_CN, 2% H_2_O, 0.1% formic acid, v/v/v) *versus* solvent A (98% H_2_O, 2% CH_3_CN, 0.1% formic acid, v/v/v) at a flow rate of 300 nl/min. The mass spectrometer was operated in a data-dependent acquisition mode. The full survey scan (*m*/*z* 400–2000) was acquired in the Orbitrap with a resolution of 30,000 at *m*/*z* 400, which was followed by five MS/MS scans in which the most abundant peptide precursor ions detected in the preceding survey scan were dynamically selected and subjected for collision-induced dissociation in the LTQ (linear ion trap) to generate MS/MS spectra (“top-5 method”).

### Quantitative analysis of antibody disulfide bond reduction by Trx

Data files of the MS runs were combined and searched against the human Swiss-Prot database with the heavy and light chain sequences of infliximab, rituximab, and trastuzumab added, using PEAKS 8 Proteomics Studio (Bioinformatics Solutions Inc.). Precursor mass tolerance was 10 ppm, and fragment ion tolerance was 0.6 Da with up to two missed trypsin cleavage sites per peptide allowed. Variable modifications were defined as deamidation on asparagine and glutamine, oxidation on methionine, and alkylation with IAA or NEM (and hydrolyzed variants) on cysteines. *de novo*, peaks-db, SPIDER, and peaks PTM algorithms were sequentially used to search against a concatenated target/decoy database, providing an empirical false discovery rate, and results are reported at a 1% target/decoy false discovery rate for both peptides and proteins.

Peptides containing IAA-labeled cysteines were selected for further analysis, ensuring that at least one cysteine from each disulfide bond was covered. Four control peptides from the heavy chains and three control peptides from the light chains, all of which contained no modifiable residues, were selected and used for normalization. All of the peptides used in the analysis for each mAb are summarized in Table S1. Precursor ion areas for the selected peptides were extracted using MS1 filtering in Skyline (57). To normalize the data, the ratio of precursor ion area of the peptide under analysis to the precursor ion areas of each of the control peptides is found for the control, Trx, and 100% reduced samples. The percentage reduction for a given cysteine is calculated using Equation 1 for each peptide in each of the biological and technical replicates.

(Eq. 1) $$\text{Percentage reduction}=\frac{\left(\left(\frac{\left(\frac{\text{Area of Cys peptide in unknown}}{\text{Area of control peptide }1\text{ in unknown}}\right)}{\left(\frac{\text{Area of Cys peptide in }100\%}{\text{Area of control peptide }1\text{ in }100\%}\right)}\times 100\right)+\left(\frac{\left(\frac{\text{Area of Cys peptide in unknown}}{\text{Area of control peptide }2\text{ in unknown}}\right)}{\left(\frac{\text{Area of Cys peptide in }100\%}{\text{Area of control peptide }2\text{ in }100\%}\right)}\times 100\right)+\cdots \left(\frac{\left(\frac{\text{Area of Cys peptide in unknown}}{\text{Area of control peptide }n\text{ in unknown}}\right)}{\left(\frac{\text{Area of Cys peptide in }100\%}{\text{Area of control peptide }n\text{ in }100\%}\right)}\times 100\right)\right)}{n}$$

From this, the mean percentage reduction and S.D. for each cysteine before and after reduction with Trx was determined.

### SEC-HPLC

SEC was performed at ambient temperature using a Dionex UltiMate 3000 UHPLC system with a Phenomenex TSK G3000SWXL, 7.8-mm inner diameter × 300-mm column (Phenomenex). Mobile phase consisted of 0.2 m sodium phosphate and 0.1 m sodium sulfate (pH 6) at a flow rate of 0.5 ml/min. 20 μg of sample reconstituted in mobile phase was injected, and the column effluent was monitored at 280 nm. Data were analyzed by NIBSC for consistency using Chromeleon software (Thermo Fisher Scientific).

### Flow cytometry

For assessing the binding of Trx-treated mAbs to their target antigen, target cells (WIL2-S for anti-CD20 mAbs, A431 for cetuximab, and BT474 for trastuzumab) were washed. For this, cells were transferred into 15-ml tubes and centrifuged at 1200 rpm. Supernatant was discarded, and 5 ml of PBS was added before centrifuging again, repeated twice. Cells were resuspended to 2 × 10^6^ cells/ml in PBS. Dilution of mAb isotype controls were prepared in PBS, and 50 μl/well was transferred into a round-bottomed plate. 50 μl (1 × 10^5^) of cells/well was added, and the plate was incubated for 15 min at RT. After washing with PBS, the cells were resuspended in 100 μl of flow buffer (0.5% BSA, 0.1% sodium azide (both from Sigma-Aldrich)) per well and stained with anti-human IgG Fc PE-conjugated antibody for 15 min at RT. Cells were washed three times with PBS and resuspended in 100 μl of flow buffer prior to analysis. Fluorescence was measured at 496 nm using a BD FACSCanto II (BD Biosciences). Data were analyzed, including PE MFI and percentage positive cells calculated as an indication of antigen expression, using FlowJo software (FlowJo, Ashland, OR).

### Reporter gene–based ADCC assay

These assays were conducted using the surrogate effector cell J9, which expresses CD16 on the surface, the ligation of which is linked to luciferase reporter gene expression. It also constitutively expresses a *Renilla* reporter gene and has been characterized in a previously published study (36). 24 h prior to the assay, J9 cells were suspended at 5 × 10^5^ cells/ml, CD20-expressing Raji suspension target cells were suspended at 5 × 10^5^ cells/ml, and the medium of HER2-expressing BT474 or EGFR-expressing A431 adherent target cells was refreshed. For the assay, 10^4^ target cells/well were washed and resuspended in complete medium with serial dilutions of antibody (doubling dilutions, starting at 500 to 1 ng/ml, equivalent to −6.3 to −9 on the log scale) for 30 min at 37 °C in a 96-well white flat-bottomed cell culture plate (Sigma-Aldrich). Each concentration was conducted in duplicate. 8 × 10^4^ J9 cells/well in complete medium were then added, and the plates were incubated at 37 °C for 18 h, after which Dual-Glo for firefly luciferase and Stop & Glo for *Renilla* luciferase, respectively, (Promega) were added according to the manufacturer's instructions to enable luminescence detection. Plates were read using a MicroBeta2 Plate counter (PerkinElmer, Beaconsfield, UK). The luciferase response was normalized by dividing the firefly luciferase signal with that of *Renilla* luciferase. Three independent assays were carried out.

### ADCC assay with lymphokine-activated killer cells

The assay was performed in LAK cell culture medium without IL-2. 1 × 10^6^ CD20-expressing Daudi, HER2-expressing BT474, or EGFR-expressing A431 target cells were loaded with 5 μm calcein acetomethoxy (AM) (Thermo Fisher Scientific) in complete medium for 30 min at 37 °C. After washing target cells in RPMI, dilutions of mAb (rituximab and ofatumumab, 1000-1 ng/ml; trastuzumab, 50,000-5 ng/ml; cetuximab, 5000-1 ng/ml) were added in equal volume to 5 × 10^4^ cells/well in round-bottomed 96-well clear plates for 30 min at 37 °C. Each concentration was conducted in duplicate. Washed NK cells were added at 5 × 10^4^ cells/well to BT474 and A431 target cells or at 2 × 10^4^ cells/well to Daudi cells at a 2:1 volume ratio. Plates were incubated for 4 h at 37 °C, after which the plate was centrifuged at 250 × *g* for 3 min. 50 μl of supernatant per well was transferred to a white 96-well flat-bottomed plate, and fluorescence was read at 490/520 nm. Target cell cytotoxicity was then determined, accounting for the spontaneous release of calcein AM from target cells, and the maximum release of calcein AM from target cells was determined by lysing the cells with lysis buffer (Thermo Fisher). Percentage of target cell cytotoxicity was calculated using Equation 2.

(Eq. 2) $$\%\text{ target cell cytotoxicity}=\frac{\left(\text{experimental value}-\text{target cells spontaneous control}\right)}{\left(\text{target cell max release}-\text{target cell spontaneous control}\right)}\times 100$$

### Degranulation of lymphokine-activated killer cells by plate bound antibody

LAK cells were added to 15-ml tubes and centrifuged at 1200 rpm for 10 min, and supernatant was discarded. To wash the cells, 5 ml of PBS was added, and the tube was centrifuged again, repeated twice. LAK cells were resuspended to 1 × 10^6^ cells/ml in LAK cell medium with 2 μm monensin (BioLegend). Plates were washed with 200 μl of PBS per well three times, and 100 μl (1 × 10^5^ cells) was added; to the positive control wells, 50 ng/ml phorbol myristate acetate and 0.5 μm ionomycin (both from Calbiochem) were added. Plates were incubated for 4 h at 37 °C. After washing three times, the cells were resuspended in 100 μl of flow buffer/well and stained with 5 μl of anti-CD107a Alexa Fluor 647 (BioLegend) for 15 min at RT. Cells were washed with PBS and resuspended in 100 μl of flow buffer prior to analysis. Fluorescence was detected at 652 nm using a BD FACSCanto II. Three independent assays were carried out.

### CD16 binding assayed by surface plasmon resonance

Experiments were performed using a Biacore T100 system (GE Healthcare, Uppsala, Sweden). Analysis temperature was set to 25 °C, and sample compartment temperature was set to 15 °C. Recombinant human CD16a extracellular domain (Val-176, C-terminal His tag), equivalent to heterozygous Fcγ receptor 3a (FcγRIIIA)158 phenylalanine/valine, was purchased from Sino Biological, Inc. The lyophilizate was reconstituted at 200 μg/ml in running buffer HEPES-buffered saline-N (HBS-N: 0.01 m HEPES, 0.15 m sodium chloride; GE Healthcare, Amersham Biosciences), aliquoted, and stored at −70 °C. Anti-histidine antibody (His capture kit, GE Healthcare) was amine-coupled on two CM5 sensor chip flow cells (Plean active ligand binding cell and a reference cell). For activation of the carboxymethyl dextran surface, coupling to reach a desired immobilization range of 6000–8000 RU, and deactivation of excess reactive groups, the following solutions were injected over the surface of the chip: 0.5 m 1-ethyl-3-(3-dimethylaminopropyl)-carbodiimide and 0.1 m *N*-hydroxysuccinimide, 25 μg/ml anti-histidine antibody, and 1 m ethanolamine-HCl, pH 8.5, all at a flow rate of 10 μl/min for 7 min. CD16a was reconstituted in running buffer (0.01 m HEPES, pH 7.4, 0.15 m NaCl, 3 mm EDTA, 0.005% (v/v) surfactant P20 (GE Healthcare, Amersham Biosciences)) to 500 ng/ml and injected for 60 s at a flow rate of 5 μl/min (in the active flow cell only). Five antibody dilutions were prepared, 2025-25 nm by diluting 3-fold, in running buffer. Starting at the lowest concentration, these were injected into both active and reference flow cells for 60 s at 30 μl/min, with a dissociation time of 60 s. The chip was regenerated using 10 mm glycine, pH 1.5, for 30 s at 30 μl/min. This cycle was repeated with the same chip for successive antibodies. Fitting of data was performed with Biacore T100 evaluation software using two-step kinetic analyses.

### CD16-binding ELISA

75 μl/well of 2 μg/ml human CD16a extracellular domain in PBS was coated onto 96-well flat-bottomed plates (Thermo Fisher Scientific) for at least 2 h at RT. Wells were washed with 200 μl of PBS Tween (0.05%) three times and blocked with 150 μl for 2 h with 5% BSA (Sigma) in PBS. mAb dilutions (4000-100 ng/ml) were prepared in PBS with 2.5% BSA and 100 μl added per well. Plates were incubated overnight at 4 °C and washed, and 100 μl of secondary antibody, anti-human IgG Fab–specific horseradish peroxidase (Sigma-Aldrich) linked at a 1:7500 dilution, 100 μl for 1 h, was added at RT. After washing, 100 μl of 3,3′,5,5′-tetramethylbenzidine substrate solution (Bio-Rad) was added, and the plates were left to develop for 10–20 min before the addition of 50 μl of H_2_SO_4_, after which absorbance was recorded at 450 nm.

### Complement-dependent cytotoxicity

1 × 10^6^ WIL2-S cells/ml were incubated with 11.2 μm calcein AM in 5% FCS RPMI for 20 min at 37 °C. Cells were washed twice before being resuspended at 2 × 10^4^ cells/well and incubated with a dilution series of antibody (50 μl, starting at 500 or 600 ng/ml) in round-bottomed 96-well clear plates for 30 min at 37 °C. Each concentration was conducted in duplicate. After the addition of Low-Tox-M rabbit complement (Cedarlane, Burlington, Canada) at a 1:10 dilution, the plate was incubated at 37 °C for 2 h. 20 min before the end of this period, lysis buffer (Thermo Fisher Scientific) was added to the “target cell maximum control.” At the end of the 2-h incubation period, the plate was processed, and target cell percentage cytotoxicity was calculated as described for the LAK cell-based ADCC assay using Equation 2. Three independent assays were carried out.

### TNF neutralization assay

100 μl of 5 × 10^4^ WEHI-164 cells/well were seeded in 96-well flat bottomed plates and incubated overnight at 37 °C. ADA and infliximab starting at 300 and 200 ng/ml, respectively, were serially diluted 1:2 in assay medium (culture medium with 1 μl/ml actinomycin D (Sigma-Aldrich); 40 IU/ml TNFα (NIBSC, international standard)). Concentrations were conducted in duplicate. Medium from cell-seeded plates was completely removed, and 100 μl of mAb dilutions were added (including a no-antibody control and TNFα control) and incubated for 18 h at 37 °C. 10 μl of CCK8 (cell-counting kit-8) solution (Sigma-Aldrich) per well was added, and the plate was incubated for 4 h at 37 °C, after which absorbance was measured at 450 nm using a SpectraMax plate reader (Molecular Devices). Three independent assays were carried out.

### Antiproliferation

100 μl of 1 × 10^4^ BT474 cells/well were seeded in 96-well flat-bottomed plates and incubated at 37 °C for 2 h. Serial dilutions of trastuzumab were made, 1:3 starting at 50 μg/ml, and each concentration was conducted in duplicate. 100 μl of mAb was added to cells and incubated at 37 °C for 5 days; a no-antibody control was also included. 25 μl of alamarBlue (Thermo Fisher Scientific) was added per well and incubated for 8 h, after which absorbance was read at 570 nm. Three independent assays were carried out.

### Statistical analysis

To assess mAb ADCC activity, mAb antigen binding, or TNF neutralization, an estimate of the EC_50_ (dose at which 50% binding or activity is observed) value was determined, as well as upper and lower 95% confidence intervals, using the four-parameter model to fit sigmoidal curves with CombiStats (European Directorate for the Quality of Medicines (EDQM), Strasbourg, France). Combistats also allowed for data analyses. EC_50_ ratios were determined, which represented the change in activity postreduction. To ensure statistical significance, homogeneity was maintained with a *p* value of >0.1 and assessed using a weighted combination.

## Author contributions

S. A. G. formal analysis; S. A. G., J. X. W., and C. M. investigation; S. A. G. and C. M. methodology; S. A. G., J. X. W., and C. M. writing-original draft; S. A. G. and C. M. writing-review and editing; M. W., I. K., J. P. D., and R. J. D. resources; M. W., R. T., I. K., J. P. D., and R. J. D. supervision; M. W., R. T., I. K., J. P. D., and R. J. D. funding acquisition; C. M. conceptualization; C. M. data curation; C. M. project administration.

## Supplementary Material

Supporting Information

## Footnotes

3 The abbreviations used are:

Trx: thioredoxin
Tr1: thioredoxin reductase 1
CV: coefficient of variation
LCL and UCL: lower and upper 95% confidence limit, respectively
GCV: geometric coefficient of variation
RA: rheumatoid arthritis
FLS: fibroblast-like synoviocyte
CHO: Chinese hamster ovary
TNF: tumor necrosis factor
IAA: iodoacetamide
SEC: size-exclusion chromatography
MFI: median fluorescence intensity
GM: geometric mean
NEM: *N*-ethylmaleimide
ADCC: antibody-dependent cellular cytotoxicity
NK: natural killer
SPR: surface plasmon resonance
LAK: lymphokine-activated killer
CDC: complement-dependent cytotoxicity
TCEP: tris(2-carboxyethyl)phosphine
FCS: fetal calf serum
DMEM: Dulbecco's modified Eagle's medium
IL: interleukin
PE: phycoerythrin
RT: room temperature
EGFR: epidermal growth factor receptor
AM: acetomethoxy.
