# Supplementary material for: The impact of thioredoxin reduction of allosteric disulfide bonds on the therapeutic potential of monoclonal antibodies
Source: J Biol Chem. 2019 Nov 14;294(51):19616–34. doi: 10.1074/jbc.RA119.010637 (PMC6926469; doi:10.1074/jbc.RA119.010637)
Supplement: Supporting Information [file supp_294_51_19616__index.html]

The impact of thioredoxin reduction of allosteric disulfide bonds on the therapeutic potential of monoclonal antibodies — Thioredoxin impacts the function of monoclonal antibodies — The impact of thioredoxin reduction of allosteric disulfide bonds on the therapeutic potential of monoclonal antibodies — Thioredoxin impacts the function of monoclonal antibodies — Supporting Information 

# The impact of thioredoxin reduction of allosteric disulfide bonds on the therapeutic potential of monoclonal antibodies

## Supporting Information

- Supporting Information (to be published online) - Supporting information
