# Supplementary material for: The impact of thioredoxin reduction of allosteric disulfide bonds on the therapeutic potential of monoclonal antibodies
Source: J Biol Chem. 2019 Nov 14;294(51):19616–34. doi: 10.1074/jbc.RA119.010637 (PMC6926469; doi:10.1074/jbc.RA119.010637)
Supplement: Supporting Information [file supp_RA119.010637_155192_1_supp_428775_q0xczc.pdf]

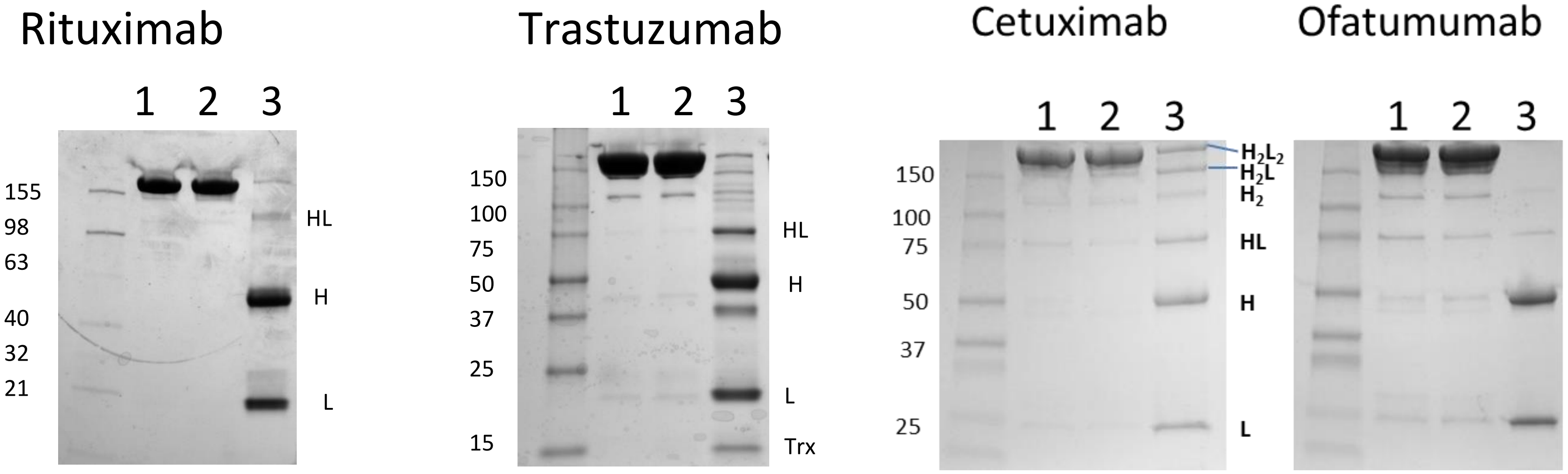

Fig. S1: Reduction of mAb interchain disulphide bonds by Trx. This was confirmed for rituximab, trastuzumab cetuximab and ofatumumab. Lane 1:no treatment; lane 2: treated with NEM only; lane 3: mAbs were incubated with Trx system for 1 hr, after which NEM was added . Washed samples were heated, run on a non-reducing SDS gel, and Coomassie staining was performed.

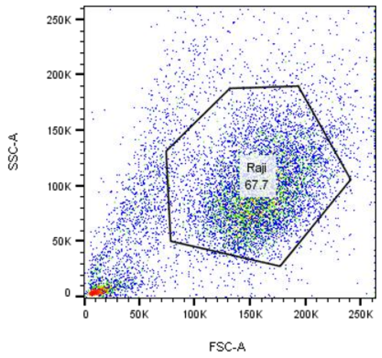

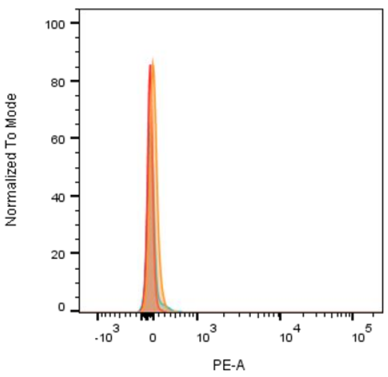

	Control sample	MFI
	Anti-IgG PE	105
	Her+ Anti-IgG PE	48
	RTX + PE Isotype	59

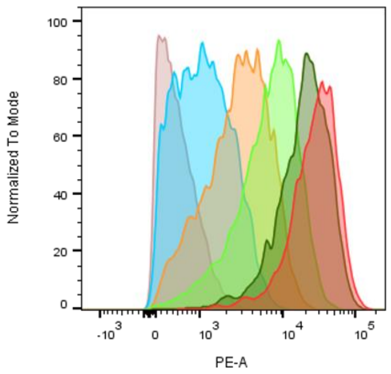

	RTX ng/ml + Anti-IgG PE	MFI
	100000	28000
	11111	20000
	1235	7000
	412	3000
	137	1100
	46	400

Fig. S2: Representative flow cytometry data for antigen binding of RITUXIMAB. CD20 expressing WIL2-S cells were incubated with different concentrations of rituximab. This was detected using a secondary PE conjugated anti-human IgG1 via flow cytometry. Controls were also set up to ensure staining to rituximab bound cells only. Data were analysed using FlowJo software, and median fluorescence intensity values (MFI) were quantified to indicate the level of secondary antibody staining. A: Representative gating of WIL2-S population to be analysed with respect to PE staining. B: Histograms representing PE staining of negative control: secondary antibody only (anti-PE IgG), 100 µg/ml anti-HER2 Trastuzumab (nonspecific for cells) with secondary antibody (Trastuzumab+ Anti-IgG PE) and 100 µg/ml rituximab with secondary PE isotype control (rituximab + PE Isotype). C: Histograms representing PE staining at different rituximab concentrations wit secondary antibody.

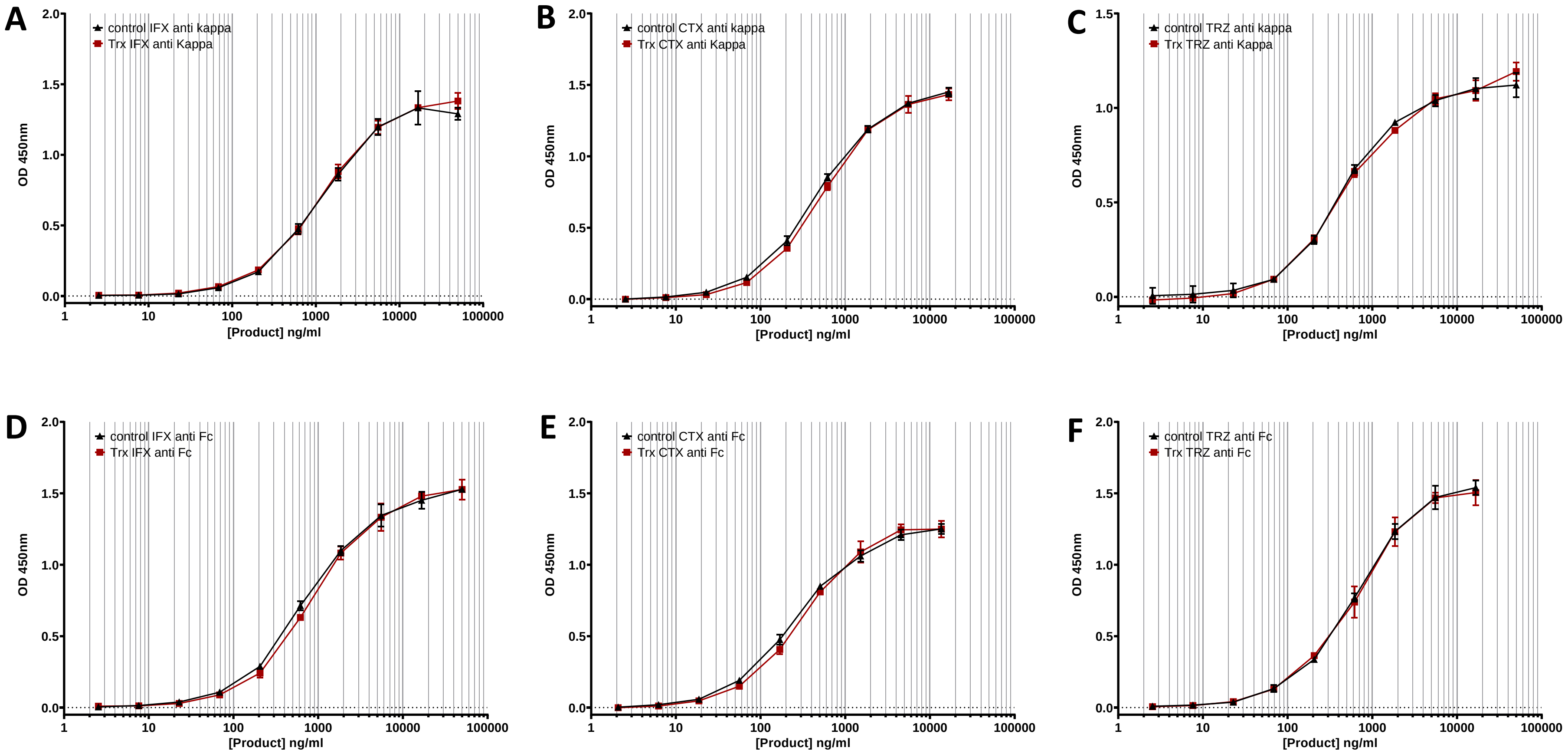

Fig S3. Trx reduction of mAbs has no effect on anti-Fc or anti-kappa secondary antibody binding. Control or Trx reduced antibody was titrated from 50,000 to 2.5 ng/ml and directly bound to ELISA plates. After blocking these were probed with either goat anti-kappa/HRP or goat anti-Fc/HRP secondary antibodies and binding levels assayed by absorbance of TMB substrate at 450nm. Representative data from three independent experiments is shown for anti-kappa/HRP binding to A) infliximab, B) cetuximab and C) Trastuzumab and for anti-Fc/HRP binding to D) infliximab, E) cetuximab and F) Trastuzumab. Each data point shows the mean and standard deviation from 4 independent readings.

Table S1: Relative EC50 data for goat anti-kappa/HRP and goat anti-Fc/HRP secondary antibodies binding to control and Trx reduced therapeutic mAbs. Data is from three independent experiments.

	**Anti-kappa/HRP**			**Anti-Fc/HRP**		
	**% binding of secondary binding to Trx reduced relative to control**	**SD**	**CV (%)**	**% binding of secondary binding to Trx reduced relative to control**	**SD**	**CV (%)**
**Infliximab**	109.97	6.56	5.96	97.93	10.17	10.38
**Cetuximab**	91.27	5.20	5.70	94.70	4.17	4.40
**Trastuzumab**	98.23	4.91	5.00	104.0	5.20	5.00

Table S2: Peptide sequences used in the quantitative mass spectrometry

**Antibody domain and Cys number**	**Peptide Sequence**
HC control	GPSVFPLAPSSK
HC control	NYYGSTYDYWGQGTTLTVSSASTK
HC control	TTPPVLDSDGSFFLYSK
HC control	GLEWVAEIR
HV-1 Cys22	LS**C**VASGFIFSNHWMNWVR
HV-1 Cys98	TEDTGVYY**C**SR
HC-1 Cys147	STSGGTAALG**C**LVK
HC-2 Cys264	TPEVT**C**VVVDVSHEDPEVK
HC-3 Cys370	NQVSLT**C**LVK
Inter-HC Cys229-Cys232	THT**C**PP**C**PAPELLGGPSVFLFPPKPK
LC control	DILLTQSPAILSVSPGER
LC control	TVAAPSVFIFPPSDEQLK
LC control	VDNALQSGNSQESVTEQDSK
LC control	DSTYSLSSTLTLSK
LV-1 Cys88	FSGSGSGTDFTLSINTVESEDIADYY**C**QQSH
LC-1 Cys134	SGTASVV**C**LLNNFYPR
LC-1 Cys194	VYA**C**EVTHQGLSSPVTK
